# N^1^-methylnicotinamide is a signalling molecule produced in skeletal muscle coordinating energy metabolism

**DOI:** 10.1038/s41598-018-21099-1

**Published:** 2018-02-14

**Authors:** Kristoffer Ström, David Morales-Alamo, Filip Ottosson, Anna Edlund, Line Hjort, Sine W. Jörgensen, Peter Almgren, Yuedan Zhou, Marcos Martin-Rincon, Carl Ekman, Alberto Pérez-López, Ola Ekström, Ismael Perez-Suarez, Markus Mattiasson, Pedro de Pablos-Velasco, Nikolay Oskolkov, Emma Ahlqvist, Nils Wierup, Lena Eliasson, Allan Vaag, Leif Groop, Karin G. Stenkula, Céline Fernandez, Jose A. L. Calbet, Hans-Christer Holmberg, Ola Hansson

**Affiliations:** 1 Lund University Diabetes Centre, Department of Clinical Sciences, Lund University, Malmö, Sweden; 2 Swedish Winter Sports Research Centre, Department of Health Sciences, Mid Sweden University, Östersund, Sweden; 3 Department of Physical Education and Research Institute of Biomedical and Health Sciences (IUIBS), University of Las Palmas de Gran Canaria, Las Palmas de Gran Canaria, Spain; 4 Department of Endocrinology (Diabetes and Metabolism), Copenhagen University Hospital, Copenhagen, Denmark; 5 Department of Medicine and Medical Specialties, Faculty of Medicine and Health Sciences, University of Alcalá, Madrid, Spain; 6 Lund University Diabetes Centre, Department of Experimental Medical Science, Lund University, Lund, Sweden; 7 Department of Endocrinology and Research Institute of Biomedical and Health Sciences (IUIBS), University of Las Palmas de Gran Canaria, Las Palmas de Gran Canaria, Spain; 8 Finnish Institute of Molecular Medicine, Helsinki University, Helsinki, Finland

## Abstract

Obesity is a major health problem, and although caloric restriction and exercise are successful strategies to lose adipose tissue in obese individuals, a simultaneous decrease in skeletal muscle mass, negatively effects metabolism and muscle function. To deeper understand molecular events occurring in muscle during weight-loss, we measured the expressional change in human skeletal muscle following a combination of severe caloric restriction and exercise over 4 days in 15 Swedish men. Key metabolic genes were regulated after the intervention, indicating a shift from carbohydrate to fat metabolism. Nicotinamide N-methyltransferase (NNMT) was the most consistently upregulated gene following the energy-deficit exercise. Circulating levels of N^1^-methylnicotinamide (MNA), the product of NNMT activity, were doubled after the intervention. The fasting-fed state was an important determinant of plasma MNA levels, peaking at ~18 h of fasting and being lowest ~3 h after a meal. In culture, MNA was secreted by isolated human myotubes and stimulated lipolysis directly, with no effect on glucagon or insulin secretion. We propose that MNA is a novel myokine that enhances the utilization of energy stores in response to low muscle energy availability. Future research should focus on applying MNA as a biomarker to identify individuals with metabolic disturbances at an early stage.

## Introduction

The number of individuals who are overweight or obese is increasing worldwide and obesity elevates the risk of many serious diseases, including certain forms of cancer, cardiovascular disease and type 2 diabetes (T2D). Failure to address a continued increase in obesity will thus have negative effects on life expectancy^1^. The total estimated cost of diabetes diagnosed in the U.S. in 2012 was $245 billion, which is an increase by more than 40% in 5 years^2^. However, obesity can be counteracted by reducing energy intake and increasing energy expenditure thereby achieving a negative energy balance. Several studies support the use of exercise in combination with dietary changes as a weight-loss strategy^3–5^. An important reason to include exercise in a weight-loss program is its effect on preserving fat-free mass (FFM)^4,6^, which is essential when combating obesity^6^. Skeletal muscle produces myokines which *e.g*. can regulate mitochondrial efficiency and thereby contribute to decreasing fat mass and insulin resistance^7^. Many weight-loss programs successfully achieve short-term weight loss, but the major challenge is that overweight/obese individuals fail to sustain the weight reduction over time^8,9^.

Most successful strategies rely on lifestyle interventions ranging from several weeks to months. However, we have recently shown that a clinically relevant reduction in fat mass can be achieved and maintained in overweight men by an intense 4-day intervention, combining prolonged low-intensity exercise (9 h/day) and severe caloric restriction^10^. Following the 4-day intervention, total body mass was reduced by 4.9 kg, of which ~40% (2.1 kg) was fat mass. More importantly, the reduction in body mass was sustained (4.4 kg) four weeks after the intervention and the relative reduction in fat mass was increased to 90% (3.8 kg) of the total decrease in body mass. The majority of this reduction (~70%) was accounted for by loss of trunk fat, leading to a diminished waist circumference (~7 cm). A significant reduction in body weight (2.4 kg) was maintained a year after the intervention, most of which (~80%) was due to loss of fat mass. This weight reduction was accompanied by improvements in blood lipids, *e.g*. reduced total cholesterol and low-density lipoprotein levels.

Although loss of adipose tissue is beneficial, a simultaneous decrease in skeletal muscle mass has negative effects on metabolism as well as muscle function and performance^11,12^. In order to minimize such negative effects of exercise during an energy deficit state, a deeper understanding on a detailed molecular level is needed. Thus, the aim of the present study was to examine changes in gene expression in skeletal muscle in response to exercise combined with severe energy deficit.

## Results

### Changes in skeletal muscle mRNA expression in response to severe energy deficit

Fifteen overweight Swedish men were exposed to a combination of caloric restriction and high-volume-low-intensity exercise for 4 days yielding a ~ 5000 kcal/day negative energy balance and an average total decrease in fat mass of 2.1 kg^10^. During the intervention, one arm was exercised each morning for 45 minutes followed by ~ 8 h of hiking. After the intervention, the participants were again examined after 3 days of isoenergetic diet and reduced exercise (maximum 10000 steps/day). Skeletal muscle biopsies were taken (3 at each time point) in the fasted state in the morning at 3 time points during the study from both the exercised and non-exercised arm muscles (*deltoid*) and from one leg muscle (*vastus lateralis*), *i.e*. before (PRE) and after (WCR, *i.e*. Walking + Caloric Restriction) the 4-day intervention and after the reexamination 3 days later (DIET). Characteristics of the participants are presented in Supplementary Table S1. To study changes in muscle gene expression in response to low energy availability, we performed a microarray analysis using the Illumina Beadarray system. During the WCR period the diet consisted of 0.8 g/kg body mass of whey protein (n = 8) or sucrose (n = 7), split into three doses (morning, midday and afternoon). There was no significant difference in muscle gene expression due to diet (whey protein or sucrose), *i.e*. no genes were observed with a significant change in expression between the two diets in response to the intervention (p_adj_ > 0.05), therefore the two diet groups were merged for subsequent analyses (n = 15). In the non-exercised arm, 39 genes were differentially expressed between PRE and WCR (false discovery rate (FDR) < 5%), 14 showed higher and 25 lower expression at WCR. In the exercised arm, 44 genes were differentially expressed (FDR < 5%), 22 genes showed higher and 22 lower expression (WCR vs. PRE, FDR < 5%). In the leg, 421 genes were differentially expressed (FDR < 5%), 207 increased and 214 decreased (WCR vs. PRE)—(Supplementary Table S2). This larger number of differentially expressed genes in the leg could reflect the higher amount of work performed by this muscle group during the intervention. The 10 most differentially expressed genes (increased and decreased) at WCR compared with PRE in the exercised, non-exercised arm and leg muscles are reported in Table 1. Genes differentially expressed in more than one tissue are presented in Supplementary Fig. S1, *e.g*. 19 genes were found in all three muscle tissues. The most consistent decreased expression was observed for the transferrin receptor (*TFRC*) gene (0.17, 0.15 and 0.19; p_adj_ < 0.001) for non-exercised, exercised arm and leg muscles respectively (WCR vs. PRE). In contrast, Nicotinamide N-methyltransferase (*NNMT*) was upregulated 5.4, 4.2 and 2.9-fold at WCR compared with PRE (p_adj_ < 0.05) in non-exercised and exercised arm and leg muscles, respectively.

**Table 1:** Top 10 genes ranked on fold change (up or down) in skeletal muscle from non-exercised arm (*deltoid*), exercised arm and leg (*vastus lateralis*) following a 4-day intervention (PRE vs. WCR).

Non-Exercised arm				Exercised arm				LEG			
Gene Symbol	Entrez GeneID	Fold change	FDR adjusted P-value	Gene Symbol	Entrez GeneID	Fold change	FDR adjusted P-value	Gene Symbol	Entrez GeneID	Fold change	FDR adjusted P-value
Top 10 up-regulated genes											
*NNMT*	4837	5.35	0.00623	*HMGCS2*	3158	5.51	0.03552	*ACTC1*	70	5.77	0.00023
*HMGCS2*	3158	4.64	0.02902	*NNMT*	4837	4.15	0.03968	*MT2A*	4502	4.47	0.00010
*PDK4*	5166	4.43	0.00526	*HMOX1*	3162	3.77	0.02117	*ANGPTL4*	51129	3.90	0.00101
*FCN3*	8547	2.87	0.01057	*ANGPTL4*	51129	3.69	0.02170	*CDKN1A*	1026	3.01	0.01522
*TPPP3*	51673	2.83	0.00085	*C13ORF39*	196541	3.67	0.01396	*NNMT*	4837	2.93	0.00851
*RHOD*	29984	2.50	0.02892	*TPPP3*	51673	2.70	0.00332	*HMOX1*	3162	2.92	0.00186
*UCP2*	7351	2.22	0.00563	*UCP2*	7351	2.66	0.00022	*SERPINA3*	12	2.81	0.00149
*GLRX*	2745	2.10	0.01057	*FCN3*	8547	2.53	0.01693	*PDK4*	5166	2.77	0.00636
*CEBPD*	1052	2.03	0.02448	*RHOD*	29984	2.38	0.04033	*LOC644150*	644150	2.71	4.30E-06
*MPP6*	51678	1.78	0.01057	*IRF7*	3665	2.25	0.00959	*FCN3*	8547	2.56	0.00016
Top 10 down-regulated genes											
*TFRC*	7037	0.17	0.00085	*TFRC*	7037	0.15	6.04E-07	*TFRC*	7037	0.19	2.56E-05
*TMEM70*	54968	0.32	0.00488	*TMEM70*	54968	0.30	0.01396	*C5ORF13*	9315	0.22	8.45E-09
*WDR62*	284403	0.34	0.00623	*G0S2*	50486	0.36	0.04413	*ITGB1BP3*	27231	0.22	0.00073
*OR7E37P*	100506759	0.37	3.54E-05	*OR7E37P*	100506759	0.39	5.06E-07	*SLC16A3*	9123	0.23	1.68E-05
*FLJ25404*	146378	0.37	0.01057	*C5ORF13*	9315	0.40	0.00311	*ZNF197*	10168	0.25	0.00023
*EXTL1*	2134	0.38	0.00488	*WDR62*	284403	0.41	0.01396	*WDR62*	284403	0.26	7.19E-05
*MASP1*	5648	0.38	0.02448	*EXTL1*	2134	0.41	0.00109	*OR7E37P*	100506759	0.30	8.45E-09
*C5ORF13*	9315	0.39	0.01057	*ATPGD1*	57571	0.45	0.00146	*ATPGD1*	57571	0.30	2.60E-07
*ATPGD1*	57571	0.43	0.00450	*CA14*	23632	0.45	0.00446	*LOC342934*	N/A	0.31	0.00074
*CA14*	23632	0.44	0.00344	*MAP6D1*	79929	0.48	0.00446	*ANK1*	286	0.31	4.15E-12

The only gene found to be differentially expressed between DIET and WCR (FDR < 5%) in the non-exercised arm was carnitine palmitoyltransferase 2 (*CPT2*). In the exercised arm, three genes, *SCHIP1*, *CPT2* and high-mobility group protein B2 (*HMGB2)*, were differentially expressed (DIET vs. WCR, FDR < 5%). Of these, *HMGB2* and *CPT2* were regulated in the opposite direction, *i.e*. increased expression after the 4-day intervention and reduced after the 3-day follow up. In the leg, 147 genes were found differentially expressed between DIET and WCR (FDR < 5%). Out of these, 121 (82%) were regulated in the opposite direction in WCR vs. PRE, *i.e*. a large proportion of the identified changes in gene expression induced by the 4-day intervention was reversed by the 3-day period with isoenergetic diet and limited exercise. All differentially expressed genes (FDR < 5%) in WCR vs. PRE and DIET vs. WCR are reported in Supplementary Tables S2 and S3, respectively.

### A severely energy deficient state leads to increased muscle expression of NNMT and elevated circulating levels of N^1^-methylnicotinamide

**Figure 1:**
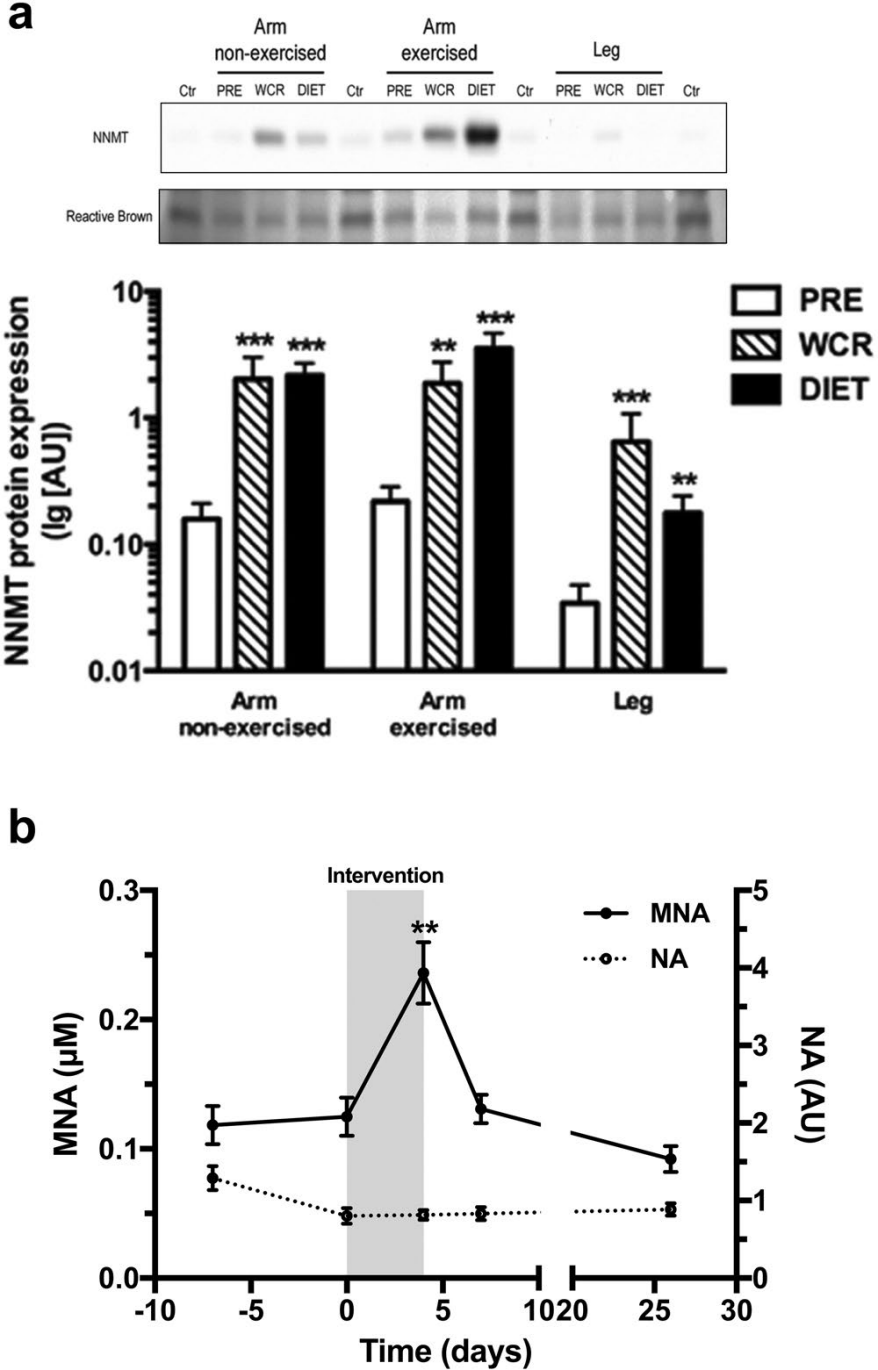
NNMT protein levels in skeletal muscle and MNA/NA plasma concentrations in humans during a 4-day low-caloric-high-volume exercise intervention. (**a**) Protein expression of NNMT in human skeletal muscle measured using Western blot before (PRE), after (WCR) and at 3 days post (DIET) a 4-day low-caloric-high-volume exercise intervention. Expression in the non-exercised arm, exercised arm and leg are given separately. A quality control sample was included on all gels (Ctr). Top square image shows a representative NNMT band (~30 kDa) (blot was cut on either side of the band before staining with antibody). Lower image is cropped from the full-length blot stained with reactive brown (for image of full-length blot, see Supplementary Fig. S6), and shows the region of the blot that was cut and stained with NNMT antibody. **p < 0.01, ***p < 0.001 using Wilcoxon signed-rank tests compared to PRE, (n = 15). No significant difference between WCR and DIET was observed. (**b**) Circulating levels of plasma nicotinamide (NA) and N1-methylnicotinamide (MNA) at five time point before and after the 4-days intervention, (n = 15). The intervention period is indicated with grey shading. Blood was drawn after 12 h of fasting at each time point. **p < 0.01 using Wilcoxon signed-rank tests compared to the average level of the two time points before the intervention. No significant difference in NA concentration was observed. Data is given as mean ± SEM.

The most consistently increased expression at WCR versus PRE was seen for the *NNMT* gene, *i.e*. the gene with the largest increase in expression in all 3 muscle tissues. The increased expression of NNMT was confirmed at the protein level using western blot with a ~12-fold (p < 0.001), ~8-fold (p < 0.01) and ~19-fold (p < 0.001) increase for non-exercised and exercised arm and leg muscles respectively (n = 15, Fig. 1a). NNMT is a methyltransferase, catalyzing N-methylation of nicotinamide (NA) to produce N^1^-methylnicotinamide (MNA) and has recently been shown to regulate energy expenditure with elevated expression in white adipose tissue (WAT) of obese and diabetic mice^13^. We therefore investigated if the severely energy deficient state, induced by the diet and exercise intervention, would result in a difference in circulating plasma levels of nicotinamide (pNA) and/or N^1^-methylnicotinamide (pMNA) using liquid chromatography coupled to mass spectrometry (LC-MS). Blood samples were drawn in the fasted state (12 h) at 5 different time points. No difference in pNA concentration was observed (Fig. 1b), but the concentration of pMNA was ~2-fold increased at WCR compared to before the intervention, *i.e*. compared to the average pMNA concentration at time points −7 and 0 days (0.24 ± 0.024 μM vs. 0.12 ± 0.010 μM, p < 0.01, n = 15, Fig. 1b).

### The nutritional state regulates pMNA levels

To test if the nutritional state alone, regardless of exercise, influences the circulating pMNA level, C57BL6/J mice were subjected to either a 4 h or 12 h fasting period. The food was withdrawn in the morning (n = 6) and one set of mice was sacrificed after 4 h (n = 4) and a second set after 12 h (n = 6). In the fed state (time = 0), the pMNA concentration was 0.15 ± 0.019 μM and increased after 4 h to 0.32 ± 0.018 (p < 0.01, Supplementary Fig. S2). After 12 h of fasting the level was 0.22 ± 0.012 μM.

**Figure 2:**
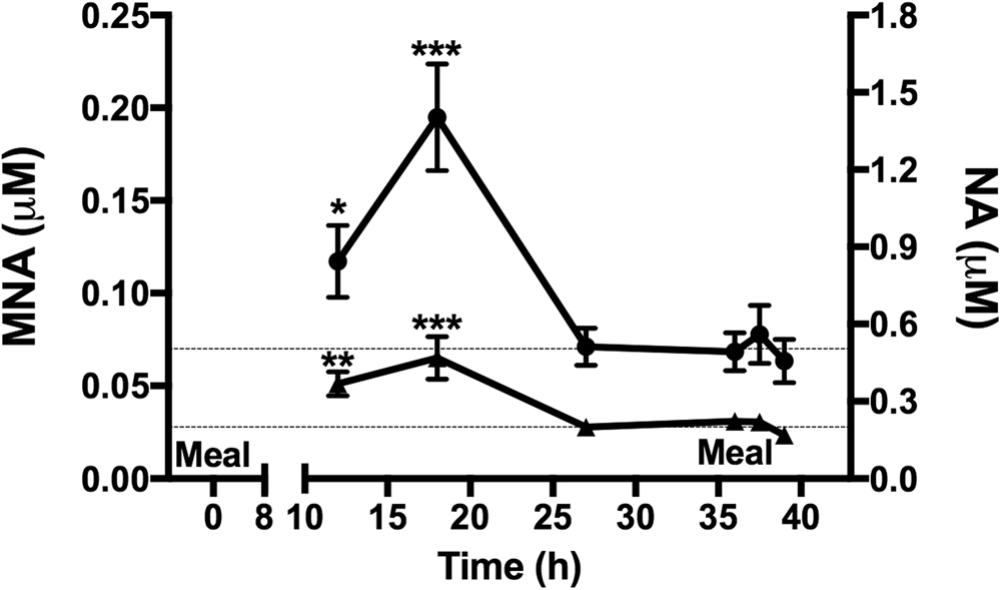
Plasma MNA and NA levels in humans during fasting and feeding. Circulating levels of plasma MNA and NA after prolonged fasting (36 h) and refeeding. *p < 0.05, **p < 0.01, ***p < 0.001 using Wilcoxon signed-rank tests. Time points 12 h, 18 h and 27 h of fasting are compared to the mean level of time points 36 h fasting, 1.5 h post feeding and 3 h post feeding, n = 18. Data is given as mean ± SEM.

To test if feeding regulates the pMNA level also in humans, 18 healthy Danish volunteers were subjected to a 36 h fast followed by a meal. The study has previously been described^14^ and characteristics of the included individuals are presented in Supplementary Table S4. Blood was drawn at 12, 18, 27 and 36 h of fasting and at 1.5 and 3 h after the meal. The pMNA level initially increased with duration of fasting (0.12 ± 0.019 μM at 12 h, p < 0.05, compared to 0.07 ± 0.009 *i.e*. the mean of [36 h, 1.5 h and 3 h post meal]), reaching a highest concentration at 18 h of fasting (0.20 ± 0.029 μM, p < 0.001, compared to 0.07 ± 0.009 *i.e*. the mean of [36 h, 1.5 h and 3 h post meal])—(Fig. 2). Extending the fasting period further led to a reduction of the pMNA concentration, reaching a lower-limit of 0.071 ± 0.0099 μM after 27 h of fasting (Fig. 2). Eating a meal after 36 h of fasting, when the pMNA concentration was 0.068 ± 0.010 μM, did not lower pMNA further (0.078 ± 0.016 μM and 0.063 ± 0.012 μM at 1.5 h and 3 h post meal, respectively, Fig. 2). In another experiment 13 healthy Danish volunteers were subjected to a 15 h fast followed by a meal. Characteristics of the participants are presented in Supplementary Table S5. Six of the volunteers participated in both experiments. After 10 h of fasting the pMNA concentration was 0.18 ± 0.063 μM. A meal, given after 15 h of fasting, yielded a lower pMNA concentration of 0.096 ± 0.016 μM 1.5 h after the meal (Supplementary Fig. S3). This level decreased further at 3 h after the meal to 0.063 ± 0.012 μM, p < 0.05 compared to 10 h of fasting (Supplementary Fig. S3).

### Increased pMNA stimulates lipolysis, but not glucagon or insulin secretion

**Figure 3:**
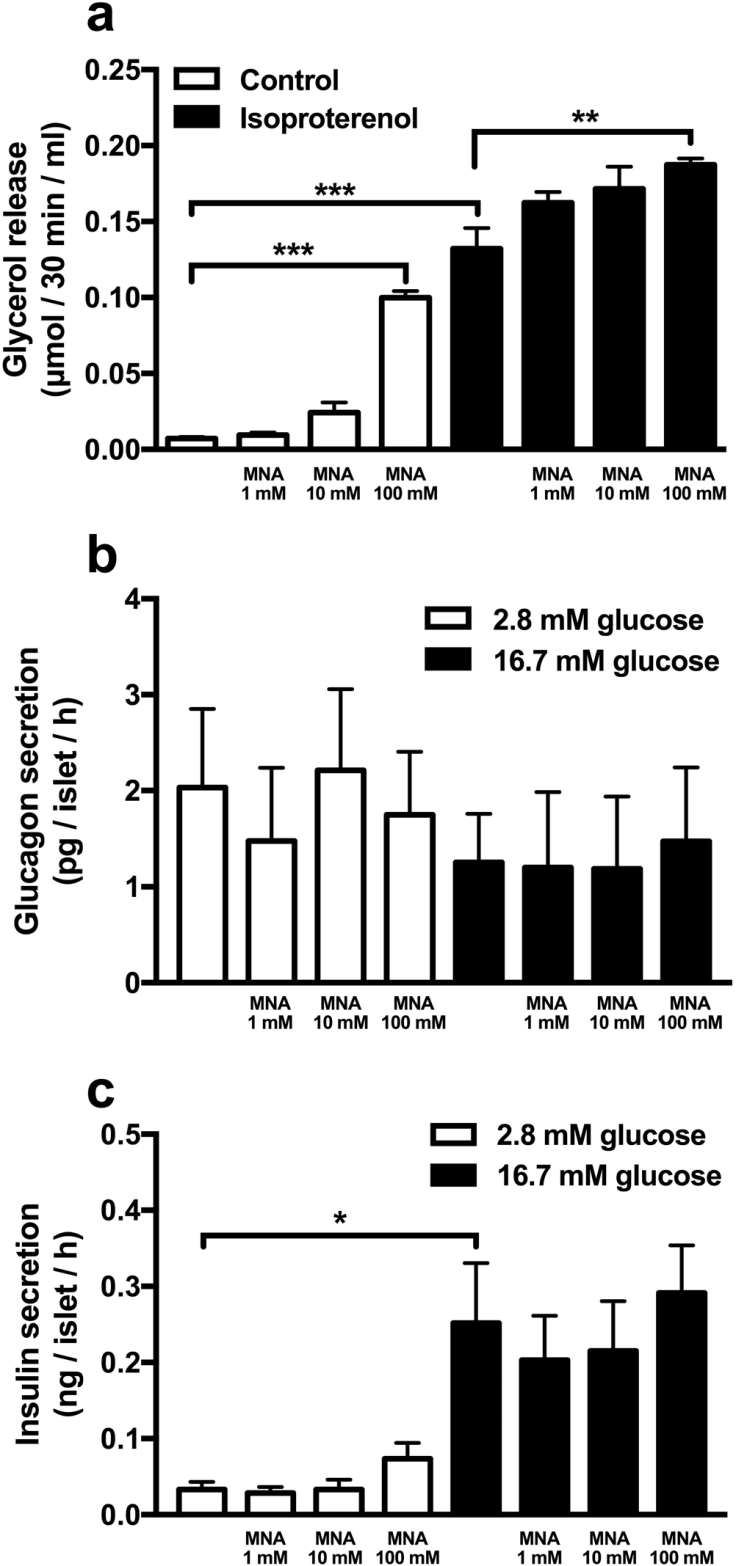
Measurements of the effect of MNA on rat lipolysis and human glucagon and insulin secretion. (**a**) Lipolysis in isolated rat adipocytes. Primary adipocytes were pre-incubated with MNA (1–100 mM) for 30 min, and then stimulated with isoproterenol (Iso, 10 nM) for additional 30 min. Glycerol release was measured in the medium both in the unstimulated and Iso-stimulated state, n = 6–10 independent experiments of pools of 2–3 rats per experiments. (**b**) Glucagon secretion from human islets of Langerhans, n = 3–5. (**c**) Insulin secretion from human islets of Langerhans, n = 4. *p < 0.05, **p < 0.01, ***p < 0.001 using Kruskal–Wallis tests with Dunn’s correction for multiple comparisons within each group (Ctrl and Iso). Data is given as mean ± SEM.

Having established an effect of energy availability on the circulating pMNA level, we hypothesized that MNA could coordinate metabolism as a cross-tissue signalling molecule able to mobilize energy substrates from stores in WAT and the liver as MNA has been shown to stimulate glucose release from isolated mouse hepatocytes^15^. An effect on lipolysis has not been shown. Therefore, we also investigated the impact of increased MNA on lipolysis in isolated primary rat adipocytes. Glycerol release was measured both at non-stimulated and isoproterenol-stimulated (10 nM) conditions with addition of 1, 10 or 100 mM MNA. In the basal state, 100 mM MNA increased glycerol release ~14-fold compared to control (0.10 ± 0.0045 vs. 0.0072 ± 0.0011 μmol/30 min/ml, p < 0.001, n = 6–10, Fig. 3a). In the isoproterenol-stimulated state, 100 mM MNA increased glycerol release ~1.5-fold compared to isoproterenol alone (0.19 ± 0.0043 vs. 0.13 ± 0.014 μmol/30 min/ml, p < 0.01, n = 6–10, Fig. 3a). Isoproterenol alone stimulated glycerol release ~18-fold (p < 0.001, n = 10, Fig. 3a). To test if MNA exerts its effects primarily on WAT and the liver or if its regulation of lipolysis and glucose production could be mediated via glucagon and/or insulin, hormone secretion was measured from human pancreatic islets of Langerhans stimulated with 1–100 mM of MNA. However, no effect of MNA on either glucagon or insulin release was found (Fig. 3b,c).

### Isolated human myotube cells have the ability to secrete MNA

**Figure 4:**
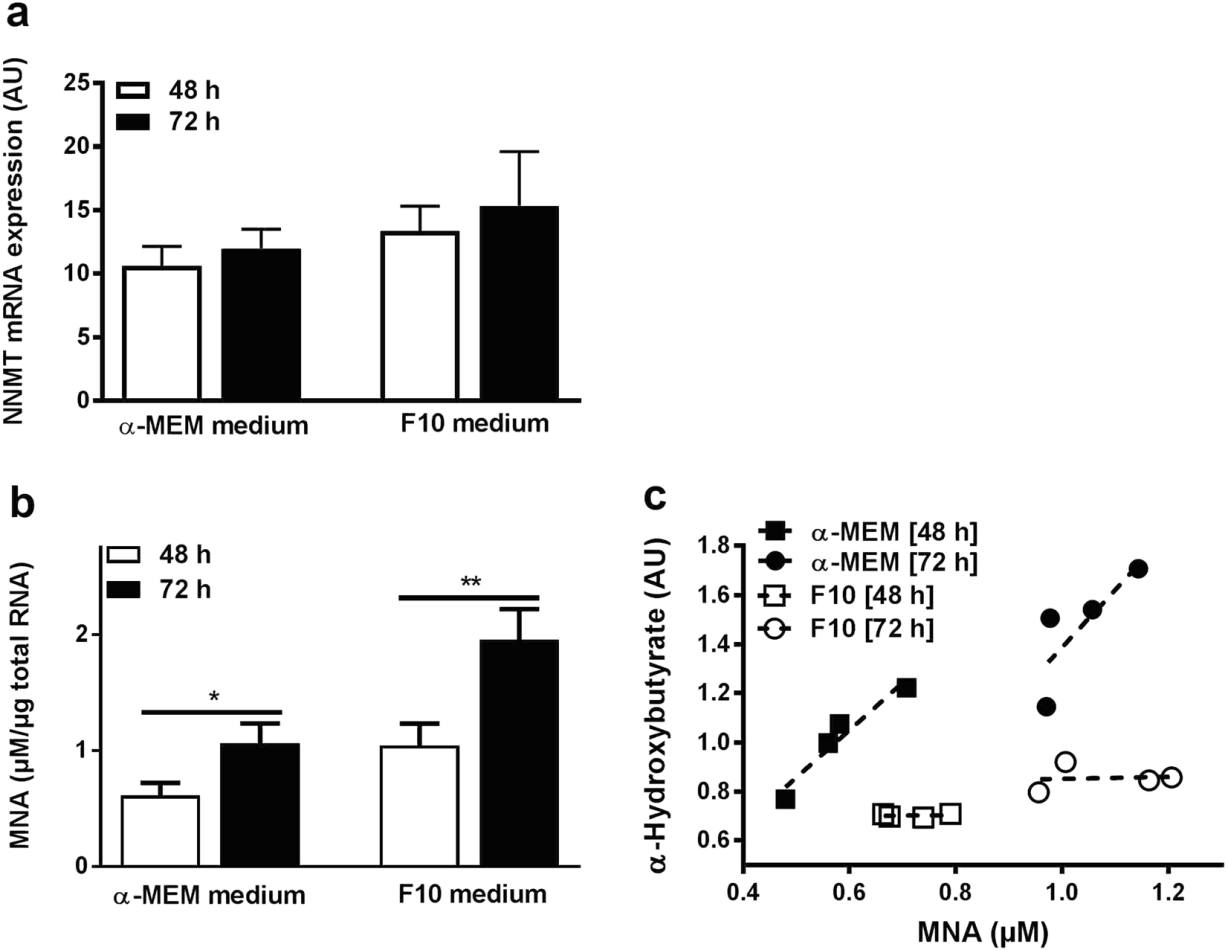
NNMT mRNA expression, and the release of MNA and α-Hydroxybutyrate from human myotubes. (**a**) The mRNA expression level of NNMT in human myotube cells measured using QPCR. The myotubes were cultured in either α-MEM or F10 medium for 48 h or 72 h. n = 5 in 1–3 experiments. (**b**) Concentration of MNA in human myotube culture medium. n = 4–5 in 1–3 experiments. The MNA concentration is expressed versus total RNA content to normalize for cell density. (**c**) Concentration of MNA and α-Hydroxybutyrate in the culture medium after 48 h or 72 h incubation in either α-MEM or F10 medium. n = 4 in 1–3 independent experiments. *p < 0.05, **p < 0.01 using Paired t tests. Data is given as mean ± SEM.

Next, we analyzed the expression of NNMT in muscle cells, and more importantly if muscle cells are able to secrete MNA. Human myotubes were cultured for 48 and 72 h in two different media, *i.e*. α-MEM or F10. Two culture conditions were chosen for replication purposes and the main difference in composition between the two media was the amount of included amino acids and NA, *e.g*. α-MEM medium contains ~1.7-fold higher NA concentration compared to F10. The mRNA expression level of NNMT and the concentration of MNA in the culture medium were measured. No difference in myotube NNMT mRNA expression was seen between the two time points (48 and 72 h) in either of the two media (10.65 ± 1.51 AU vs. 11.99 ± 1.53 AU [α-MEM] and 13.39 ± 1.94 vs. 15.34 ± 4.28 [F10], or between the two media at either time point (10.65 ± 1.51 AU vs. 13.39 ± 1.94 [48 h] and 11.99 ± 1.53 AU vs. 15.34 ± 4.28 [72 h], n = 5 in 1–3 independent experiments, Fig. 4a). However, the concentration of MNA in the medium was increased by 1.7-fold and 1.9-fold at 72 h vs. 48 h in both α-MEM medium (1.06 ± 0.17 vs. 0.61 ± 0.11 μM/μg RNA_tot_, p < 0.05, n = 5 in 1–3 independent experiments) and in F10 medium (1.95 ± 0.27 vs. 1.04 ± 0.19 μM/μg RNA_tot_, p < 0.01, n = 5 in 1–3 independent experiments), respectively (Fig. 4b). Interestingly, increased levels of MNA was observed in F10 compared to α-MEM medium, showing a 1.8-fold increase at 72 h of culture (1.95 ± 0.27 vs. 1.06 ± 0.17 μM/μg RNA_tot_, p = 0.056 with a Mann-Whitney U test, n = 5 in 1–3 independent experiments, Fig. 4b), indicating that the concentration of amino acids may influence muscle MNA secretion. No difference in the concentration of NA was seen between the two time points in either medium (Supplementary Fig. S4). To screen for metabolites correlating with the release of MNA, we measured the concentration of 47 metabolites in the media using LC-MS after culturing human myotubes as described above, *i.e*. with combination of different durations (48 and 72 h) and media (α-MEM and F10) (Supplementary Fig. S5). 2-Hydroxybutanoic acid (*i.e*. α-Hydroxybutyrate, α-HB) was the most correlated metabolite with MNA in α-MEM medium (α-MEM: r^2^ = 0.93 [48 h] and 0.64 [72 h]; F10: r^2^ = 0.009 [48 h] and 0.010 [72 h] (Fig. 4c)).

## Discussion

In this study, we examined changes in skeletal muscle gene expression after caloric restriction in combination with an exercise intervention. The expression of key genes involved in metabolic regulation (*e.g. CPT2*, *PDK4* and *PFKFB3*) was altered after the 4-day intervention, indicating a shift from carbohydrate to fat metabolism. As expected, we observed an increased expression of *PDK4* after the 4-day intervention, indicating a low carbohydrate oxidation rate, as PDK4 is an inhibitor of the pyruvate dehydrogenase complex (PDC) and thereby the transport of pyruvate into the mitochondria. An increased lipid oxidation rate was also supported by the increased expression of *CPT2*, an important part of the transport system of fatty acids into the mitochondria. The results also indicate increased muscle wasting, *i.e*. increased expression of genes involved in the ubiquitine proteasome system (*e.g. TRIM63* and *PSMD4*), as well as in the autophagy-lysosomal proteolytic system (*e.g. BNIP3*, *CTSD* and *SQSTM1*). A majority of the changes observed were however reversed by 3 days of isoenergetic diet and limited exercise.

The major finding was that the expression of *NNMT* in skeletal muscle was consistently increased after the intervention with a concomitant increase of pMNA. In connection, nicotinamide riboside kinase 2 (ITGB1BP3) and poly [ADP-ribose] polymerase 1 (PARP1), proteins involved in the NAD^+^-salvage pathway, were found to have lower expression after the intervention. Fasting alone increased the pMNA concentration and eating a meal, when pMNA was high, reduced pMNA to approximately 0.07 μM after 3 h. Interestingly, while the concentration of NA was not significantly changed at the severely energy deficient state (Fig. 1b), a concomitant increase in NA was seen with the increase of MNA in response to fasting alone (Fig. 2). NNMT has recently been shown to regulate energy expenditure with elevated expression in WAT in obese and diabetic mice^13^. Knockdown of NNMT in WAT and liver in mice protects against diet-induced obesity by increasing cellular energy expenditure. However, these experiments were not performed in tissue-specific mouse models and a possible effect of skeletal muscle NNMT knockdown was not considered^13^. Chronic MNA treatment lowers fasting glucose levels and prolongs the survival of rats with streptozotocin-induced diabetes^16^. Patients with T2D show approximately two fold higher expression of NNMT in WAT compared to healthy controls^17^ and elevated levels of MNA both in the circulation^17,18^ and in urine^19^. The pMNA level is positively correlated with BMI^17,19^, inversely with insulin sensitivity^17^ and bariatric surgery has been shown to reduce both WAT NNMT expression and the level of pMNA^17^. In liver, NNMT is a positive regulator of gluconeogenesis, via a sirtuin 1-dependent mechanism and MNA stimulates gluconeogenesis in primary hepatocytes^15^. In addition, here we show that MNA also has the ability to stimulate lipolysis in isolated rat adipocytes, but has no effect on the release of insulin or the starvation hormone glucagon from human islets of Langerhans. Out of the 47 measured metabolites in media from cultured human myotubes, the MNA concentration was most strongly correlated with that of α-HB. α-HB has been suggested to be an early biomarker of insulin resistance and a predictor of glucose intolerance^20,21^. The results presented here clearly demonstrate that the NNMT-MNA axis plays an important role as a regulator of energy metabolism, supported by previous studies^13,15,17^. In addition, data presented in literature^13,16–19^ also show that a perturbation of this mechanism is seen in obesity and T2D. Taken together, these results suggest that pMNA could be a potential biomarker for metabolic flexibility^22,23^ and possibly be used as an early marker for metabolic disease.

Here we show an increase of muscle NNMT protein and pMNA concentrations after exercise and caloric restriction. It has previously been reported that a 12-week exercise intervention led to a reduction of WAT *NNMT* expression in prediabetic and diabetic individuals, but no change in healthy controls^17^. The effect on pMNA was not measured. In contrast, a single bout of endurance exercise increased liver NNMT activity and pMNA^24^. However, the increased pMNA level observed after exercise could not be explained by the regulation of liver NNMT, as liver NNMT activity was not increased after exercise in IL-6 knockout mice, but yet led to an increase of pMNA^24^. Although adipocytes have been shown to secrete MNA^25^, the increased pMNA seen after exercise cannot be explained by expressional regulation of *NNMT* in WAT or the liver. The data presented here suggest that this elevated pMNA concentration could be explained by release of pMNA from skeletal muscle under exercise and/or fasting conditions. However, in the metabolically unhealthy, *i.e*. obese, prediabetic or T2D states, a correlation between WAT *NNMT* expression and the fasting pMNA level have been described^17^, indicating that a dysregulation of the NNMT-MNA axis in WAT may play a greater role under such conditions. How regulation of muscle NNMT activity influences metabolism in prediabetic and diabetic individuals remains to be studied, as all individuals examined here were non-diabetic. However, the duration of fasting should be considered in all comparisons, as our results showed an initial increase and subsequent decrease in pMNA levels after fasting.

Caloric restriction and weight reduction have many positive effects on metabolism and also increase liver *NNMT* expression in mice^15,26^. Here we show a similar regulation of *NNMT* expression in human skeletal muscle. This is in contrast to WAT *NNMT* expression, which is reduced by weight reduction^17^. Thus, the regulatory mechanisms of *NNMT* expression as well as the mechanisms underlying the downstream effects are most likely different in these tissues. In line with the regulation in liver^15^, we postulate that the downstream effects of muscle NNMT activity are not mediated by a shift in the methyl donor balance, as in WAT^13^, but rather by its product MNA. The muscle energy-sensing mechanism is however unclear, but possibly involves a signal emanating from the balance between anaerobic and aerobic metabolism, or a shift from carbohydrate to lipid oxidation. Caloric restriction elicits increased activity of proteins in the sirtuin family (*e.g*. Sirt1)^27^, which in turn results in lysine deacetylation and breakdown of nicotinamide adenine dinucleotide (NAD+) to NA and 1′-O-acetyl-ADP-ribose^28^ or 2′- and 3′-O-acetyl-ADP-ribose^29^. An increased activity of NNMT during such low-energy states could serve to maintain a high degree of deacetylase activity and fat oxidation by removing NA and thereby its feedback-inhibition of Sirt1^30^. NNMT may thus serve as a sensor for the change in the redox system of the cell. The level of circulating amino acids could possibly play a part in the muscle energy-sensing mechanism, as indicated by the experiments in myotubes showing higher concentration of MNA in the amino acid poor F10 medium (Fig. 4b).

In summary, we have shown that a high-volume-low-intensity exercise intervention in combination with caloric restriction elevates the expression of *NNMT* in human skeletal muscle, and that this increase is mirrored by a rise in the fasting level of pMNA. The pMNA level can also be regulated by the nutritional state alone, *i.e*. fasting leads to an initial increase followed by a decline, while eating a meal when the pMNA level is high lowers this level. MNA can be secreted from human myotubes and stimulates lipolysis, but has no effect on glucagon or insulin release. On the basis of these findings and the existing literature, we propose that MNA is a myokine that signals directly to WAT and the liver to mobilize energetic substrates when the availability of energy is low in muscle. Future research should focus on applying MNA as a biomarker for skeletal muscle insulin sensitivity to identify individuals with metabolic disturbances at an early stage.

## Methods

### Participants

The Ostersund study^10^ was designed to get insight into the mechanisms that regulate muscle metabolism, muscle mass and fat mass during severe energy deficit in humans. To isolate the potential effects due to exercise from those elicited by the neuro-endocrine systemic adaptations, muscle biopsies were obtained from three different skeletal muscles subjected to different levels of exercise. The inclusion criteria were: 1) an age of 18–55 years, 2) stable body weight for at least 3 months prior to the start of the experiments, 3) a BMI ≥ 25 kg/m^2^, 4) a waist circumference >102 cm, and 5) 20–40% body fat. The exclusion criteria were: 1) orthopedic limitations incompatible with prolonged walking or exercise, 2) smoking and 3) chronic disease of any kind. Sixteen men were recruited initially, but one retired due to incompatibility with his working duties. The 15 remaining participants were assigned randomly to a diet consisting of either sucrose (n = 7) or whey protein (n = 8) during study phase III. Characteristics of participants are presented in Supplementary Table S1. The study was divided into five different phases: I = baseline assessment (PRE), II = intervention (WCR), III = recovery (DIET), IV = follow up 4 weeks post intervention (4W) and V = follow up 1 year post intervention (1Y). The 4-day intervention during phase II consisted of walking, arm cranking exercise + caloric restriction. The 3-day recovery during phase III consisted of isocaloric diet and reduced exercise (maximum 10000 steps per day). During phase I and at the end of phases II, III and IV, body composition (Lunar iDXA, GE Healthcare, Madison, WI; and Bioimpedance, InBody 720, Biospace Co. Ltd, Seoul, Korea) and VO_2max_ using a metabolic cart (Jaeger Oxycon Pro, Viasys Healthcare, Hoechberg, Germany) during an incremental exercise test to exhaustion on a cycle ergometer (Monark 839E, Ergomedic, Sweden) were assessed. In addition, during phases I-IV 30-ml blood samples were drawn, following a 12 h overnight fast. In phase I, blood samples were obtained on two occasions, one week apart. During phase V, only body composition was assessed. A full description of the experimental protocol can be found elsewhere^10^.

The 36 h fasting study has previously been published^14^. Briefly, 18 men (age = 24.6 ± 1.2 years and BMI = 22.9 ± 3.2 kg/m^2^) were recruited from the Danish National Birth Registry as healthy, non-diabetic, with no diabetes in two family generations. Characteristics of the participants are presented in Supplementary Table S4. All were subjected to 36 h of fasting and sampling. 8–16 weeks after the 36 h fasting study, 6 of these subjects participated in a second study together with 7 other matched males born with a low birth weight. Characteristics of the participants are presented in Supplementary Table S5. The 13 participants received a control diet and were subjected to a 15 h fast. During both study settings, the participants were allowed ad-libitum water. For 72 h prior to the study interventions, the participants received a control diet of precooked meals for standardization of energy intake (10 MJ per day, 50% carbohydrate, 35% fat, 15% protein). Furthermore, the participants were not allowed to perform exercise or consume alcohol or soft drinks in these three days. All blood samples were immediately distributed into tubes, placed on ice, and centrifuged (Eppendorf Centrifuge 5810R, Eppendorf AG, Hamburg, Germany). Plasma was obtained and stored at −80 C until later analysis.

### Microarray analysis of muscle biopsies

Fifteen overweight Swedish men participated in a 4-day intervention combining severe caloric restriction with prolonged exercise, as previously described. Muscle biopsies were obtained from right and left *deltoid* muscle and from *vastus lateralis* of either leg. RNA was extracted from 10–20 mg of muscle biopsies using a TissueLyser II (Qiagen) and the RNeasy Fibrous Tissue mini kit (Qiagen). RNA concentration was estimated using a NanoDrop ND-1000 spectrophotometer (A260/A280 > 1.8 and A260/A230 > 1.0 (NanoDrop Technologies, Wilmington, DE, USA). RNA integrity was evaluated using the 2100 Bioanalyzer Instrument (Agilent Technologies). Input RNA, 100 ng for the leg and 200 ng for the arm samples, was used for subsequent biotinylation and hybridization. TotalPrep™-96 RNA Amplification Kit (Thermo Fisher Scientific) was used to generate biotinylated, amplified cRNA for subsequent hybridization of 750 ng (150 ng/µl) on the Human HT-12 v4 Expression BeadChip (Illumina) and scanning on the iScan system (Illumina). The raw data was read and quantified using Illumina Genome Studio version 1.1.1. Summary level expression data was read using the Bioconductor beadarray package. Differential expression analysis was performed using the Bioconductor limma package. P-values were corrected using the False Discovery Rate method, with an acceptance of < 0.05.

### Total protein extraction, electrophoresis, and Western blot analysis

Protein extracts from the muscle biopsies were prepared as previously described^31^, and total protein content was quantified using the bicinchoninic acid assay^32^. Briefly, proteins were solubilized in sample buffer containing 0.0625 M Tris-HCl (pH 6.8), 2.3% (wt/vol) sodium dodecyl sulfate (SDS), 10% (vol/vol) glycerol, 5% (vol/vol) β-mercaptoethanol, and 0.001% (wt/vol) bromophenol blue. The total protein to load and the optimal antibody concentration was determined by linear regression from a gradient of protein extracts at concentrations ranging from 20 to 45 μg. The linear relation between total protein concentration loaded and quantitative intensity of the bands was calculated. After confirming linearity in this range, equal amounts (30 μg) of each sample were electrophoresed with 10% SDS-PAGE as previously described^33^ and transferred to an Immun-Blot PVDF Membrane for Protein Blotting (Bio-Rad Laboratories). To control for differences in loading and transfer efficiency across membranes, membranes were stained with Reactive Brown 10 (0.07%) and destained with water for 10 sec to remove background. Loading was similar for control and experimental samples (data not shown). After this, membranes were destained for 3 × 10 min in NaOH (0.1 N). To determine NNMT total protein amount, the membrane, cut on either side of the region for the NNMT band (~30 kDa), was incubated with an antibody directed against NNMT (NNMT (G-4), a mouse monoclonal antibody raised against NNMT of human origin, sc-376048, Santa Cruz Biotechnology), diluted (1:1000) in 4% BSA, in Tris-buffered saline with 0.1% Tween 20 (TBS-T; BSA-blocking buffer). Antibody-specific labelling was revealed by incubation with goat anti-mouse IgG-HRP (sc-2031, Santa Cruz Biotechnology), diluted (1:5000) in 5% blocking buffer, and visualized with the ClarityTM Western ECL Substrate kit (Bio-Rad Laboratories) using the ChemiDoc XRS system (Bio-Rad Laboratories) and analysed with Quantity One (Bio-Rad Laboratories). Densitometry analyses were carried out immediately. Samples from each subject were run on the same gel. In addition, in all gels, a 30 μg control sample obtained from the extraction of 5 g of pooled human skeletal muscle from different healthy donors was loaded 4 times per gel as an internal control.

### Measurements of pMNA and NA using LC-MS

Measurements of 1-methylnicotinamide were performed in human serum, mouse EDTA plasma and cell media using a UHPLC-QTOF-MS System (Agilent Technologies 1290 LC, 6550 MS, Agilent Technologies, Santa Clara, CA, USA). All samples were stored at −80 °C and thawed on ice before metabolite extraction was performed by addition of six volumes of extraction solvent, consisting of 80:20 LC-MS grade methanol/water containing the stable isotope labelled 1-methylnicotinamide-d4 (Toronto Research Chemicals Inc., Toronto, Canada). Samples were incubated at 4 °C, with mixing at 1250 rpm during 1 hour, before precipitated proteins were removed by centrifugation for 20 minutes at 14000 × g. The supernatant was transferred to glass vials. Sample preparation for measurement of α-HB in cell media was performed in the same manner. Liquid chromatography separation was performed by injecting 2 µl sample on an Acquity UPLC BEH Amide column (1.7 μm, 2.1 * 100 mm; Waters Corporation, Milford, MA, USA) or on an ACE C18 column (1.7 μm, 2.1 * 100 mm; Advanced Chromatography Technologies Ltd., Aberdeen, UK) for measurements of 1-methylnicotinamide and α-HB respectively. Separation on Acquity UPLC BEH Amide was performed by gradient elution using two mobile phases (mobile phase A: H_2_O with 10 mM ammonium formate and 0.1% formic acid; mobile phase B: acetonitrile with 0.1% formic acid) using a flow rate of 0.4 ml/min and subsequent mass spectrometric analysis was performed in positive electrospray ionization. Gradient elution on ACE C18 (mobile phase A: H_2_O with 0.1% formic acid; mobile phase B: acetonitrile with 0.1% formic acid) was performed with at flow rate of 0.3 ml/min and subsequent mass spectrometric analysis utilized negative electrospray ionization. The auto sampler was kept at 16 °C. The sheath gas temperature was set at 350 °C and the sheath gas flow at 12 l/min. The drying gas flow was 14 l/min and was delivered at 200 °C. Mass spectra were acquired at a rate of 1 spectra/s and the mass range was 70–1000 m/z (mass-to-charge ratio). Samples were analysed in batches of maximum 45 samples, where pooled QC samples were injected every 5 samples and in the beginning of each batch to ensure high repeatability and to condition the LC-column respectively.

### Adipocyte isolation and lipolysis assay

To measure lipolysis, rat adipocytes were isolated from epididymal fat tissue, as described previously^34^. Cells were suspended (10% suspension) in Krebs-Ringer (KRH) medium containing 25 mM Hepes pH 7.4, 200 nM adenosine and 1% BSA (w/v), preincubated with MNA (1–100 mM) for 30 min, and then stimulated with isoproterenol (Iso, 10 nM) for additional 30 min as indicated in the figures (at 37 °C, with shaking, 150 cycles/min). After 30 min, samples were placed on ice for 20 min, and 200 µl of the cell medium was subsequently removed for enzymatic determination of the glycerol content, as described previously^35^.

### Glucagon and insulin secretion measurements in isolated human islets of Langerhans

Islets from 6 human donors (gender = F3/M3, age 55 ± 7, BMI 26.5 ± 2.0 kg/m^2^, HbA1c 5.9 ± 0.1, days in culture 2.8 ± 0.6) were provided by the Nordic Network for Clinical Islet Transplantation (Uppsala, Sweden). Human islets were hand-picked to ensure high purity. Insulin and glucagon secretion was measured in static batch incubations described previously^36^. Briefly, batches with 12 islets (in quadruplicates) were pre-incubated in Krebs Ringer solution (120 mM NaCl, 4.7 mM KCl, 2.5 mM CaCl_2_2H_2_O, 1.2 mM KH_2_PO_4_, 1.2 mM MgSO_4_, 10 mM HEPES, 25 mM NaHCO_3_, pH 7.4, supplemented with carbogen and 1 mg/ml of bovine serum albumin) supplemented with 1 mM glucose for 30 min, followed by 1 h incubation at 2.8 and 16.7 mM glucose in absence or presence of MNA (1–100 mM) as indicated. Insulin secretion was measured using radioimmunoassay kit (Euro-Diagnostica, Malmö, Sweden) and glucagon secretion was measured with ELISA (Mercodia, Uppsala, Sweden).

### Culturing of human myotubes

Human primary Skeletal Muscle Derived Cells (SkMDC) from five different donors were purchased from Cook Myosite (Pittsburg, US). The myoblast cells were cultured and differentiated into myotubes following instructions supplied by the vendor. Briefly, cells were maintained in growth basal medium with growth supplements (MK-2288; Cook Myosite), 10% fetal calf (FCS) serum (Thermo Fisher Scientific) and antibiotics (0.4% penicillin/streptomycin (P/S). Cells were passaged at ~50% confluence. To induce differentiation, cells were plated at ~45000 cells/cm^2^ in 6-well plates. Growth media was replaced with differentiation media (MD-9999; Cook Myosite) with 2% FCS and 0.4% P/S, and the cultures were incubated for 3 days, during which time myotube differentiation occurred (monitored using the expression of differentiation markers, *i.e*. Myosin Heavy Chain 2 and Myocyte-specific enhancer factor 2C). After differentiation, the multinucleated myotubes were either cultured in α-Minimum Essential Medium (α-MEM) or Ham’s F10 Nutrient Mix (F10) (Thermo Fisher Scientific; catalog numbers 22571 and 31550 respectively) for 48 h or 72 h, containing 2% FCS and 0.4% P/S. At each time point, total RNA was extracted from the cell cultures.

### Quantitative real-time PCR

RNA was isolated from the muscle cells using the RNeasy Fibrous Tissue Mini Kit (Qiagen), and DNA was removed on-column using the RNase-Free DNase Set (Qiagen). Concentration and purity was measured using a NanoDrop ND-1000 spectrophotometer (A_260_/A_280_ > 1.8 and A_260_/A_230_ > 1.0) (NanoDrop Technologies, USA). RNA integrity was verified using the 2200 TapeStation instrument (Agilent Technologies, CA, US). Reverse transcription was performed on 0.2 µg RNA using the QuantiTect reverse transcription kit (Qiagen). QPCR was carried out using an ABI 7900HT sequence detection system with 10 ng cDNA in 10 µl reactions and TaqMan Expression PCR Master Mix with duplex assays according to the manufacturer’s recommendations (Applied Biosystems, Thermo Fisher Scientific). All samples for each gene were analyzed in triplicates on the same 384 well plate (maximum accepted standard deviation in Ct-value of 0.1 cycles) with 3 endogenous controls (*POLR2A*, *HPRT1* and *CYCA*). Probe sets used: *NNMT* (Hs00196287_m1). Endogenous control probe sets: *POLR2A* (Hs00172187_m1), *HPRT1* (4326321E, VIC-MGB) and *CYCA* (4326316E, VIC-MGB).

### pMNA in mice during fasting

Male C57BL/6J mice (Taconic, Ry, Denmark) were used at 10–12 weeks of age. Animals were on a 12 h light cycle with non-restricted food (chow) and water. Food was withdrawn in the morning and blood was sampled through Vena Saphena for serum analysis following 0, 4 or 12 h of fasting (n = 4–6 animals/time point). Each animal was sacrificed following blood sampling.

### Statistical analysis

Differences between group medians were analyzed using Mann-Whitney U tests or Kruskal-Wallis test with Dunn’s correction for multiple comparisons where appropriate. Analysis of paired data, *i.e*. repeated measures on the same sample, were performed using Wilcoxon signed-rank test. Correlations were calculated using Spearman’s method. In all tests p < 0.05 was considered statistically significant. Data was analyzed with SPSS 19.0 (SPSS, Chicago, IL, USA) and Graphpad 7 (Graphpad software, La Jolla, CA, USA).

### Accordance

The methods were carried out in accordance with the relevant guidelines and regulations.

### Study approval and informed consent

The Ostersund study was approved by the Regional Ethical Review Board of Umeå University (Umeå, Sweden), as well as the ethical committee of the University of Las Palmas de Gran Canaria (Canary Islands, Spain). All participants provided written consent after being informed about potential risks and benefits, prior to inclusion in the study.

The study designs and protocols in the 36 h and 15 h fasting studies were approved by the local ethics committee and applied to the Helsinki II declaration. Informed consent was obtained from the participants prior to the study.

The local ethics committee in Lund/Malmö approved the use of material from deceased human donors for the glucagon and insulin secretion measurements in isolated human islets of Langerhans, and all animal experiments.

### Data availability

The datasets generated during and/or analysed during the current study are available from the corresponding author on reasonable request.

## Electronic supplementary material

Supplementary Information

Supplementary Table S2

Supplementary Table S3

## References

[1] Stewart ST, Cutler DM, Rosen AB (2009). Forecasting the effects of obesity and smoking on U.S. life expectancy. N Engl J Med.

[2] Association AD (2013). Economic costs of diabetes in the U.S. in 2012. Diabetes Care.

[3] Shaw, K., Gennat, H., O’Rourke, P. & Del Mar, C. Exercise for overweight or obesity. *Cochrane Database Syst Rev*, CD003817, 10.1002/14651858.CD003817.pub3 (2006). 10.1002/14651858.CD003817.pub3 PMC9017288 17054187

[4] Washburn RA (2014). Does the method of weight loss effect long-term changes in weight, body composition or chronic disease risk factors in overweight or obese adults? A systematic review. PLoS One.

[5] Foster-Schubert KE (2012). Effect of diet and exercise, alone or combined, on weight and body composition in overweight-to-obese postmenopausal women. Obesity (Silver Spring).

[6] Weinheimer EM, Sands LP, Campbell WW (2010). A systematic review of the separate and combined effects of energy restriction and exercise on fat-free mass in middle-aged and older adults: implications for sarcopenic obesity. Nutr Rev.

[7] Fisher FM (2012). FGF21 regulates PGC-1α and browning of white adipose tissues in adaptive thermogenesis. Genes Dev.

[8] Wing RR, Hill JO (2001). Successful weight loss maintenance. Annu Rev Nutr.

[9] Riebe D (2005). Long-term maintenance of exercise and healthy eating behaviors in overweight adults. Prev Med.

[10] Calbet JA, Ponce-González JG, Pérez-Suárez I, de la Calle Herrero J, Holmberg HC (2015). A time-efficient reduction of fat mass in 4 days with exercise and caloric restriction. Scand J Med Sci Sports.

[11] Carbone JW, McClung JP, Pasiakos SM (2012). Skeletal muscle responses to negative energy balance: effects of dietary protein. Adv Nutr.

[12] Zibellini, J. *et al*. Effect of diet-induced weight loss on muscle strength in adults with overweight or obesity—a systematic review and meta-analysis of clinical trials. *Obes Rev*, 10.1111/obr.12422 (2016). 10.1111/obr.12422 27126087

[13] Kraus D (2014). Nicotinamide N-methyltransferase knockdown protects against diet-induced obesity. Nature.

[14] Jørgensen SW (2015). Metabolic response to 36 hours of fasting in young men born small vs appropriate for gestational age. Diabetologia.

[15] Hong S (2015). Nicotinamide N-methyltransferase regulates hepatic nutrient metabolism through Sirt1 protein stabilization. Nat Med.

[16] Watała C (2009). Anti-diabetic effects of 1-methylnicotinamide (MNA) in streptozocin-induced diabetes in rats. Pharmacol Rep.

[17] Kannt A (2015). Association of nicotinamide-N-methyltransferase mRNA expression in human adipose tissue and the plasma concentration of its product, 1-methylnicotinamide, with insulin resistance. Diabetologia.

[18] Liu M (2015). Serum N(1)-Methylnicotinamide Is Associated With Obesity and Diabetes in Chinese. J Clin Endocrinol Metab.

[19] Salek RM (2007). A metabolomic comparison of urinary changes in type 2 diabetes in mouse, rat, and human. Physiol Genomics.

[20] Gall WE (2010). alpha-hydroxybutyrate is an early biomarker of insulin resistance and glucose intolerance in a nondiabetic population. PLoS One.

[21] Ferrannini E (2013). Early metabolic markers of the development of dysglycemia and type 2 diabetes and their physiological significance. Diabetes.

[22] Felber JP, Vannotti A (1964). Effects of Fat Infusion on Glucose Tolerance and Insulin Plasma Levels. *Medicina experimentalis*. International journal of experimental medicine.

[23] Kelley DE, Mandarino LJ (2000). Fuel selection in human skeletal muscle in insulin resistance: a reexamination. Diabetes.

[24] Chłopicki S (2012). Single bout of endurance exercise increases NNMT activity in the liver and MNA concentration in plasma; the role of IL-6. Pharmacol Rep.

[25] Riederer M, Erwa W, Zimmermann R, Frank S, Zechner R (2009). Adipose tissue as a source of nicotinamide N-methyltransferase and homocysteine. Atherosclerosis.

[26] Estep PW, Warner JB, Bulyk ML (2009). Short-term calorie restriction in male mice feminizes gene expression and alters key regulators of conserved aging regulatory pathways. PLoS One.

[27] Nemoto S, Fergusson MM, Finkel T (2004). Nutrient availability regulates SIRT1 through a forkhead-dependent pathway. Science.

[28] Tanner KG, Landry J, Sternglanz R, Denu JM (2000). Silent information regulator 2 family of NAD- dependent histone/protein deacetylases generates a unique product, 1-O-acetyl-ADP-ribose. Proc Natl Acad Sci USA.

[29] Jackson MD, Denu JM (2002). Structural identification of 2′- and 3′-O-acetyl-ADP-ribose as novel metabolites derived from the Sir2 family of beta -NAD+-dependent histone/protein deacetylases. J Biol Chem.

[30] Gerhart-Hines Z (2007). Metabolic control of muscle mitochondrial function and fatty acid oxidation through SIRT1/PGC-1alpha. EMBO J.

[31] Guerra B (2007). Leptin receptors in human skeletal muscle. J Appl Physiol (1985).

[32] Smith PK (1985). Measurement of protein using bicinchoninic acid. Anal Biochem.

[33] Laemmli UK (1970). Cleavage of structural proteins during the assembly of the head of bacteriophage T4. Nature.

[34] Rodbell M (1964). METABOLISM OF ISOLATED FAT CELLS. I. EFFECTS OF HORMONES ON GLUCOSE METABOLISM AND LIPOLYSIS. J Biol Chem.

[35] Zmuda-Trzebiatowska E, Oknianska A, Manganiello V, Degerman E (2006). Role of PDE3B in insulin-induced glucose uptake, GLUT-4 translocation and lipogenesis in primary rat adipocytes. Cell Signal.

[36] Edlund A, Esguerra JL, Wendt A, Flodström-Tullberg M, Eliasson L (2014). CFTR and Anoctamin 1 (ANO1) contribute to cAMP amplified exocytosis and insulin secretion in human and murine pancreatic beta-cells. BMC Med.

