# Supplementary material for: N1-methylnicotinamide is a signalling molecule produced in skeletal muscle coordinating energy metabolism
Source: Sci Rep. 2018 Feb 14;8:3016. doi: 10.1038/s41598-018-21099-1 (PMC5813101; doi:10.1038/s41598-018-21099-1)
Supplement: Supplementary file 1 — Supplementary Information [file 41598_2018_21099_MOESM1_ESM.pdf]

Supplementary Information

N^1-methylnicotinamide is a signalling molecule produced in skeletal muscle coordinating energy metabolism

Kristoffer Ström[1, 2], David Morales-Alamo [3], Filip Ottosson[1], Anna Edlund[1], Line Hjort[4], Sine W. Jörgensen[4], Peter Almgren[1], Yuedan Zhou[1], Marcos Martin-Rincon[3], Carl Ekman[1], Alberto Pérez-López[3, 5], Ola Ekström[1], Ismael Pérez-Suárez[3], Markus Mattiasson[6], Pedro de Pablos-Velasco[7], Nikolay Oskolkov[1], Emma Ahlqvist[1], Nils Wierup[1], Lena Eliasson[1], Allan Vaag[4], Leif Groop[1, 8], Karin G. Stenkula[6], Céline Fernandez[1], Jose A. L. Calbet[3], Hans-Christer Holmberg[2]*, and Ola Hansson[1]*

[1] Lund University Diabetes Centre, Department of Clinical Sciences, Lund University, Malmö, Sweden

[2] Swedish Winter Sports Research Centre, Department of Health Sciences, Mid Sweden University, Östersund, Sweden

[3] Department of Physical Education and Research Institute of Biomedical and Health Sciences (IUIBS), University of Las Palmas de Gran Canaria, Las Palmas de Gran Canaria, Spain

[4] Department of Endocrinology (Diabetes and Metabolism), Copenhagen University Hospital, Copenhagen, Denmark

[5] Department of Medicine and Medical Specialties, Faculty of Medicine and Health Sciences, University of Alcalá, Madrid, Spain

[6] Lund University Diabetes Centre, Department of Experimental Medical Science, Lund University, Lund, Sweden

[7] Department of Endocrinology and Research Institute of Biomedical and Health Sciences (IUIBS), University of Las Palmas de Gran Canaria, Las Palmas de Gran Canaria, Spain

[8] Finnish Institute of Molecular Medicine, Helsinki University, Helsinki, Finland

Supplementary Figures

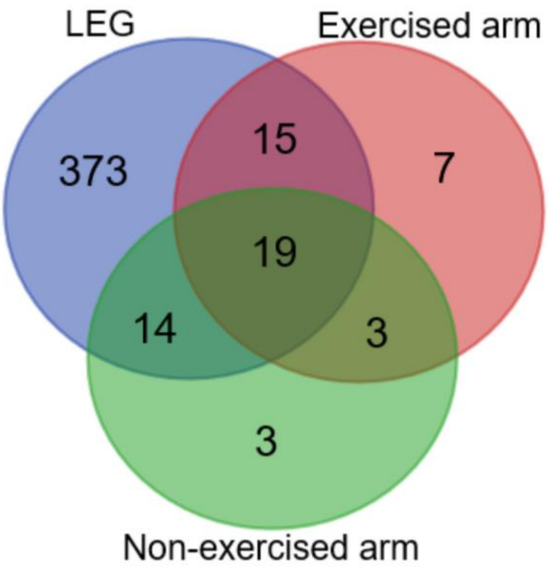

Tissues with differentialy expressed genes	Total number of genes	Genes
Exercised arm<br>LEG<br>Non-exercised arm	19	EXTL1 RHOD LOC390557 GADL1 LDHA FCN3 CA14 C5ORF13 TPPP3 CCPG1 OR7E37P ASS1 TFRC DLEU1 WDR62 NNMT GLRX ATPGD1 NOL3
Exercised arm<br>LEG	15	HMOX1 ANGPTL4 IRF7 MAP6D1 GPX3 TRIM7 HS.553217 HEXB C13ORF39 CHMP1B ANK1 HMGB2 YPEL3 STAT5A CPT2
LEG<br>Non-exercised arm	14	PKM2 CCDC69 CEBPD DNAJB5 FLJ25404 PPM1J LOC642005 MASP1 TPI1 SLC38A3 MPP6 PDK4 TP53INP2 SCHIP1
Exercised arm<br>Non-exercised arm	3	HMGCS2 TMEM70 UCP2

Supplementary Fig. S1. Genes differentially expressed at PRE versus WCR

The number and gene symbol names of genes found differentially expressed before (PRE) versus after (WCR) the 4-day intervention of caloric restriction and high-volume-low-intensity exercise in more than one tissue.

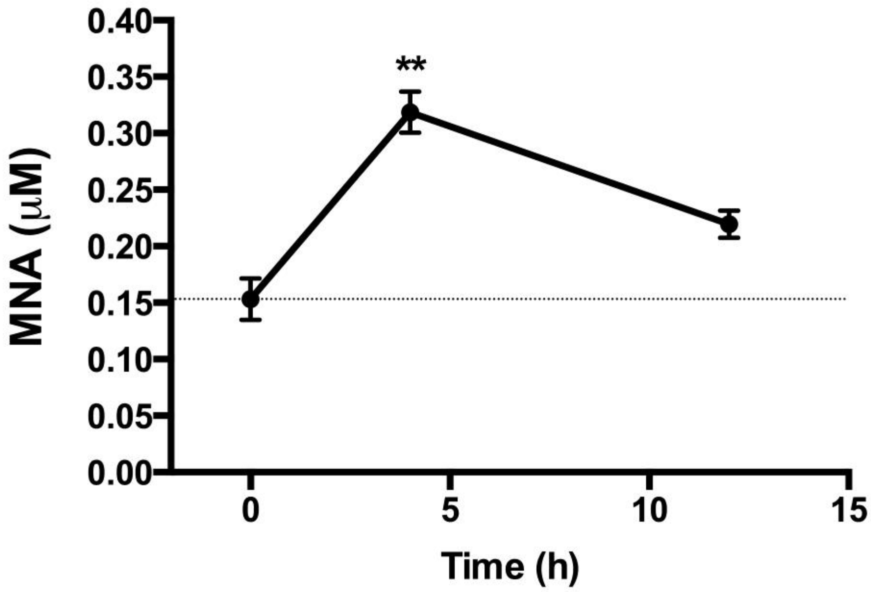

Supplementary Fig. S2. Plasma N1-methylnicotinamide (MNA) levels in mice during fasting and feeding

Circulating levels of plasma MNA after 4 and 12 h fasting in mice. ** $p < 0.01$ using Kruskal–Wallis tests with Dunn´s correction for multiple comparisons versus fed (time = 0). n = 4 – 6. Data is given as mean ± SEM.

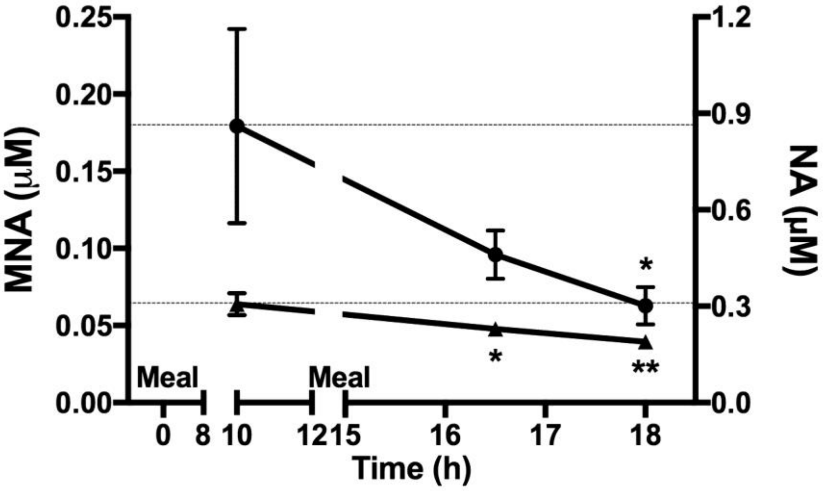

Supplementary Fig. S3. Plasma MNA and NA levels in humans during fasting and feeding

Circulating levels of plasma MNA and nicotinamide (NA) after 15 h of fasting and refeeding. * $p < 0.05$, ** $p < 0.01$ using Wilcoxon signed-rank tests. Time points 1.5 h and 3 h post feeding is compared to 10 h of fasting, n = 13. Data is given as mean ± SEM.

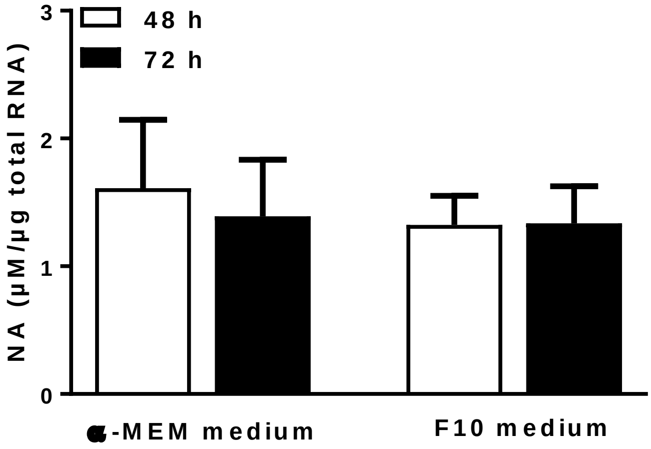

Supplementary Fig. S4. Nicotinamide (NA) release from human myotubes.

Concentration of NA in human myotube culture medium. n = 5 in 1-3 experiments. The NA concentration is expressed versus total RNA content to normalize for cell density. Data is given as mean ± SEM.

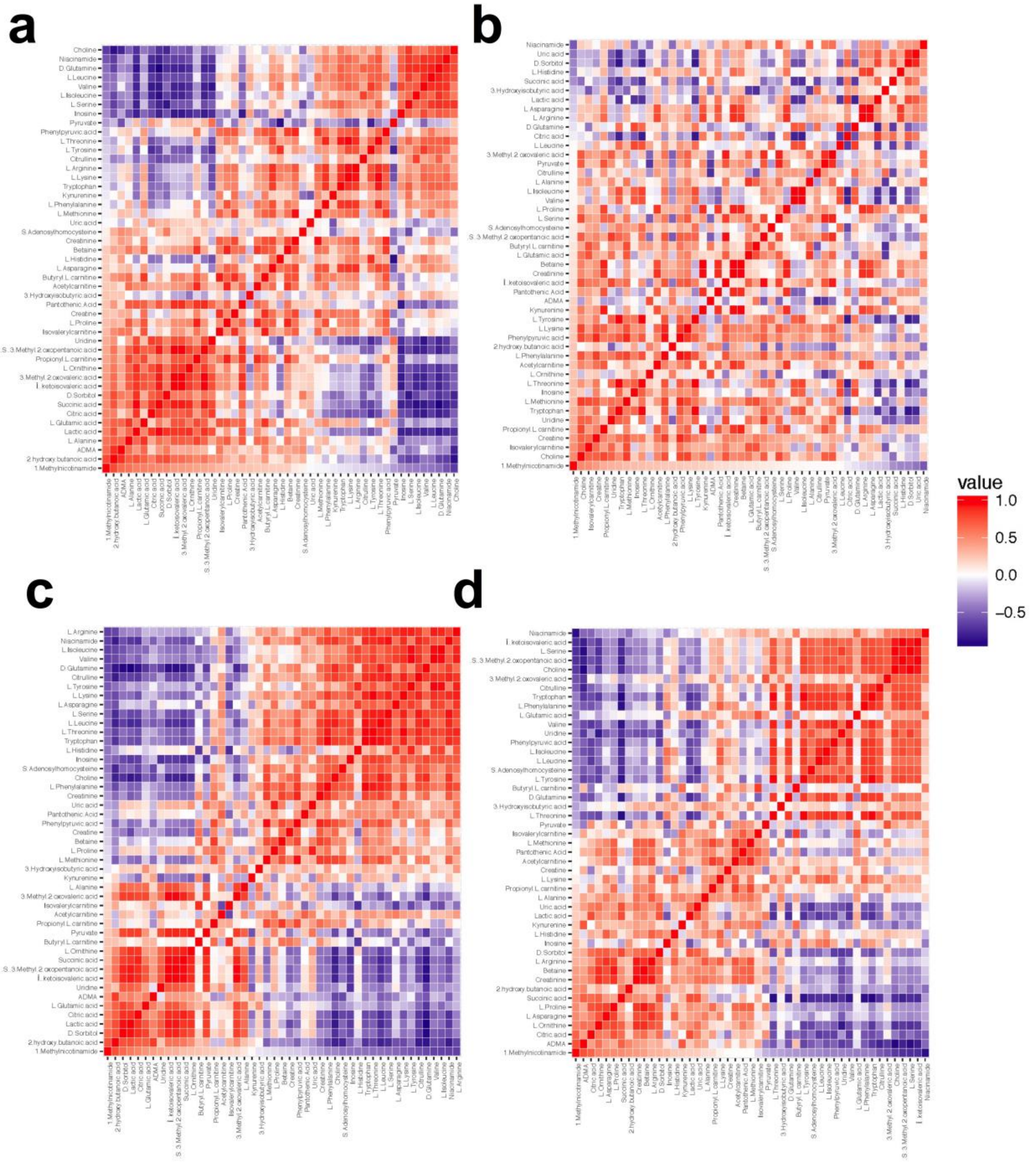

Supplementary Fig. S5. Correlation matrix of 47 metabolite levels in human myotubes culture media

Human myotubes cultured in α-MEM (**a** and **c**) or F10 (**b** and **d**) media for 48 h (**a** and **b**) or 72 h (**c** and **d**). The correlation coefficients are displayed in colour code with red = positive and blue = negative correlation. The average of n = 4 in 1-3 experiments are shown.

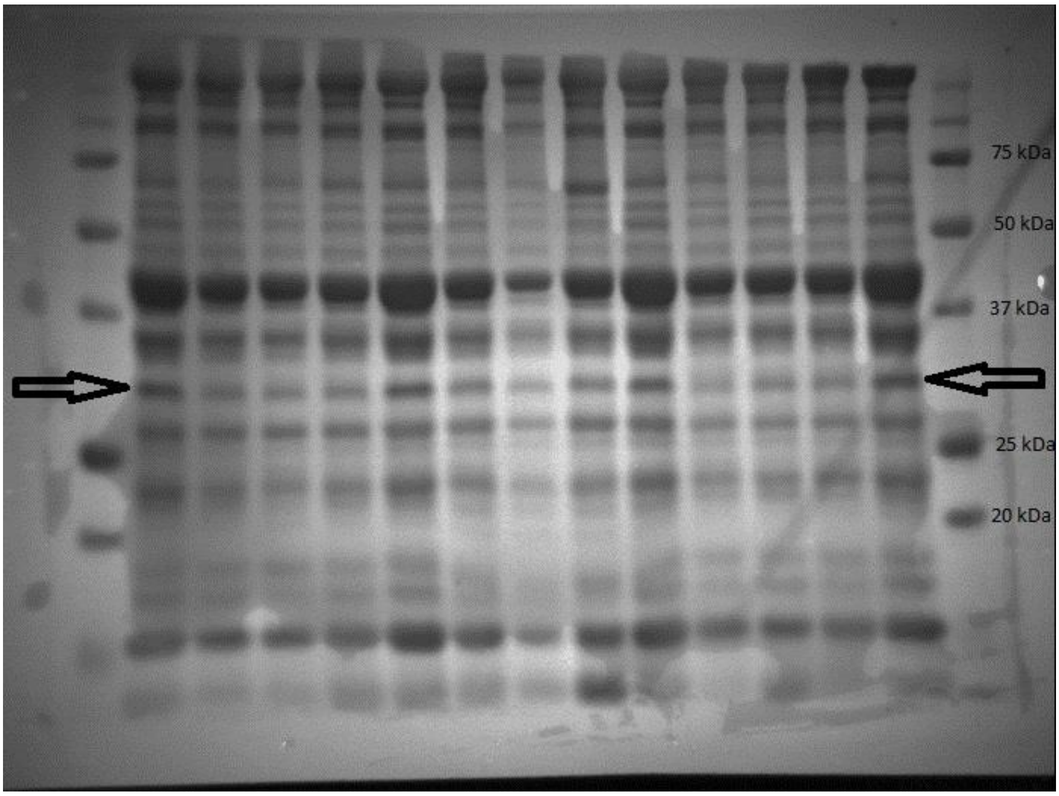

Supplementary Fig. S6. Full unedited blot for Figure 1a stained with Reactive Brown

To control for differences in loading and transfer efficiency across membranes, membranes were stained with Reactive Brown 10. Arrows indicate region (~30kDa) that was cut and stained with NNMT antibody.

Supplementary Tables

Supplementary Table S1

The pretest physical characteristics (means ± SD) of the participants of the exercise and caloric restriction intervention (n = 15).

	DIET	
	Sucrose (n=7)	Whey protein (n=8)
Age (years)	38.7 ± 8.2	43.0 ± 8.0
BMI (kg/m^2)	29.9 ± 3.1	30.9 ± 4.2
Lean mass (kg)	63.1 ± 3.1	65.4 ± 6.0
Fat mass (kg)	31.5 ± 9.1	31.4 ± 9.2
Body fat (%)	31.6 ± 5.3	30.9 ± 4.1
RMR (kcal/day)	1780 ± 272	1970 ±348
VO_{2max} (mL/kg/min)	38.8 ± 6.0	39.7 ± 5.5
Systolic BP (mmHg)	135 ± 7	132 ± 13
Diastolic BP (mmHg)	88 ± 5	89 ± 9

Abbreviations:
BMI, Body Mass Index; RMR, Resting Metabolic Rate; BP, Blood Pressure

Supplementary Table S4

Physical characteristics of the included individuals in the 36h fasting study (means ± SD) (n=18).

Age (years)	24.6 ± 1.20
BMI (kg/m^2)	22.9 ± 3.23
Lean mass (kg)	65.3 ± 7.03
Fat mass (kg)	13.4 ± 5.46
Body fat (%)	16.5 ± 4.77
RMR (kcal/day)	1681 ± 146

Abbreviations:
BMI, Body Mass Index; RMR, Resting Metabolic Rate; BP, Bood Pressure

Supplementary Table S5

Characteristics of the included individuals in the 15h fasting study (means ± SD) (n=13).

Age (years)	25.3 ± 1.21
BMI (kg/m^2)	22.0 ± 3.31
Lean mass (kg)	63.0 ± 4.41
Fat mass (kg)	11.3 ± 4.57
Body fat (%)	14.7 ± 4.43
RMR (kcal/day)	1548 ± 155

Abbreviations:
BMI, Body Mass Index; RMR, Resting Metabolic Rate; BP, Bood Pressure
